# The relationship between internal and external loads as a tool to monitor physical fitness status of team sport athletes: a systematic review

**DOI:** 10.5114/biolsport.2022.107021

**Published:** 2021-08-27

**Authors:** Adriano Lima-Alves, João Gustavo Claudino, Daniel Boullosa, Crislaine Rangel Couto, Francisco Teixeira-Coelho, Eduardo M. Pimenta

**Affiliations:** 1 School of Physical Education, Physiotherapy and Occupational Therapy, Universidade Federal de Minas Gerais, Sports Department, Belo Horizonte, Minas Gerais, Brazil; 2 Associação Chapecoense de Futebol, Santa Catarina, Brazil; 3 School of Physical Education and Sport, Universidade de São Paulo, Laboratory of Biomechanics, São Paulo, São Paulo, Brazil; 4 LOAD CONTROL, Research and Development Department, Contagem, Minas Gerais, Brazil; 5 Instituto Integrado de Saúde, Universidade Federal de Mato Grosso do Sul, Campo Grande, Brazil; 6 Sport and Exercise Science, James Cook University, Townsville, Australia; 7 iLOAD Solutions, Brasília, Brazil; 8 Centro Universitário Metodista Izabela Hendrix Universidade Federal de Minas Gerais; 9 Universidade Federal do Triângulo Mineiro, Departamento de Ciências do Esporte, Instituto de Ciências da Saúde, Uberaba, MG, Brasil

**Keywords:** Efficiency performance index, Load control, Fatigue, Physical test, Effindex

## Abstract

The efficiency index (Eff_index_) combines internal and external loads, and it has been considered a promising tool to evaluate physical fitness status. However, its real applicability and limitations have not been elucidated yet. To examine and discuss the findings from studies that used Eff_index_ as a tool for the evaluation of physical fitness status in team sports. A systematic search was performed using the Preferred Reporting Items for Systematic Reviews and Meta-Analyses (PRISMA). The databases conferred were PubMed, Scopus, Web of Science, SPORTDiscus, MEDLINE and CINAHL. The articles selected were published up to March 2021. Fourteen articles were included after meeting the inclusion criteria. A wide variety of combinations of external and internal loading parameters to calculate Eff_index_ were found. The pooled sample included 349 male participants (23 ± 3 years). Fifty-nine percent of the sample were soccer players, 20% rugby players, 10% Australian football players, 7% hurling players, and 4% basketball players. Most Eff_index_ calculations used total distance (TD) divided by heart rate derived parameters. However, recent studies have suggested the use of accelerations as the external load parameter. Eff_index_ is a simple and powerful tool for the evaluation of physical fitness status in team sports athletes. The disparity of external and internal loading parameters used to calculate Eff_index_ may affect its sensitivity to detect changes in fitness status in different team sport settings. More studies with indoor team sports and female athletes are warranted.

## INTRODUCTION

The internal-to-external load concept has been well recognised to identify the quality and quantity of training loads in competitive sport [1–3]. The external load refers to the physical work executed during the training and internal load refers to the psychophysiological responses occurring during the execution of the exercise [2, 3]. Thus, the internal load experienced from a specific external load can change according to fitness status [3] and the combination between external and internal loads can serve to assess the physical fitness of an athlete during a specific exercise. Nowadays, the assessment of internal and external loads is very common in team sports, due to the easy use of monitoring technologies such as the Global Positioning System (GPS) and heart rate (HR) monitors [4–8]. Training monitoring is an important process to manage injury risk, to assess fatigue and the associated need for recovery and to avoid the risk of negative training adaptations [1, 2]. Therefore, the control of internal and external load variables has attracted the attention of practitioners for training monitoring of elite team sports.

In the context of team sports, internal and external loads have been related through an internal-to-external load ratio. This ratio has been called the efficiency index (Eff_index_) [7, 9] and there are different ways to calculate it. For example, meters per min (m·min^-1^) and total distance (TD)/average percentage of maximal heart rate (%HR_max_) have been used in elite soccer [7, 9], m·min^-1^/training impulse (TRIMP)[10] has been used in amateur soccer, and m·min^-1^/session rating of perceived session (sRPE) has been used in Australian football [11]. More recently, another study showed higher validity when using an acceleration parameter for Eff_index_ calculation (i.e. TD × acceleration/%HR_max_)[12].

Eff_index_ has been evaluated and compared with other performance parameters in the literature [13]. In soccer and rugby athletes, Eff_index_ decreased over the match [9, 14], which suggests that Eff_index_ would be related to increased fatigue during matches [15]. In amateur soccer players, an association has been demonstrated between Eff_index_ and the anaerobic threshold [10], thus highlighting the relationship between physical fitness status and Eff_index_. Additionally, it has been suggested that Eff_index_ could be a tool to assess physical fitness status when an athlete is covering a certain distance for a given cardiovascular demand [2], because submaximal HR is inversely associated with improvements of physical fitness [16]. One recent narrative review discussed the role of Eff_index_ in the sport context and, despite the limitations of HR [17] and sRPE [2], concluded that Eff_index_ could be a useful training monitoring tool in team sports.

Since there is no great difficulty to obtain Eff_index_ data, this training monitoring tool can help to obtain more information about an athlete’s physical fitness status, which, in turn, could contribute to better management of training loads. Assuming that external load in team sports may increase due to both physical and contextual factors (e.g. quality of the opponent and season period) [18], it can be hypothesized that the Eff_index_ may be less influenced by contextual factors for evaluating physical fitness. In addition, due to very frequent congested schedules, high travel demands, and excess of accumulated fatigue during the competitive season, Eff_index_ may also be more attractive to evaluate team sports athletes than standard maximal and submaximal physical tests [19, 20].

The number of studies using Eff_index_ in team sports has been rapidly growing over the last years [12, 13, 21]. However, researchers have presented different correlation magnitudes between Eff_index_ and fitness parameters, ranging from small to large [10, 13, 22]. These findings could be a consequence of 1) different exercise protocols used, 2) different competitive levels of athletes, and 3) different calculation methods. Thus, it is not clear 1) which Eff_index_ calculation is more appropriate, 2) how this ratio could be objectively applied, and 3) whether Eff_index_ could be used in any context. Due to the increased importance of Eff_index_ as a promising monitoring tool, its better understanding through the current systematic review could be helpful for scientists and conditioning staff. Therefore, the purpose of this systematic review was to examine and discuss the studies using Eff_index_ as a tool for the evaluation of physical fitness in team sports.

## MATERIALS AND METHODS

### Search strategy

**FIG. 1:**
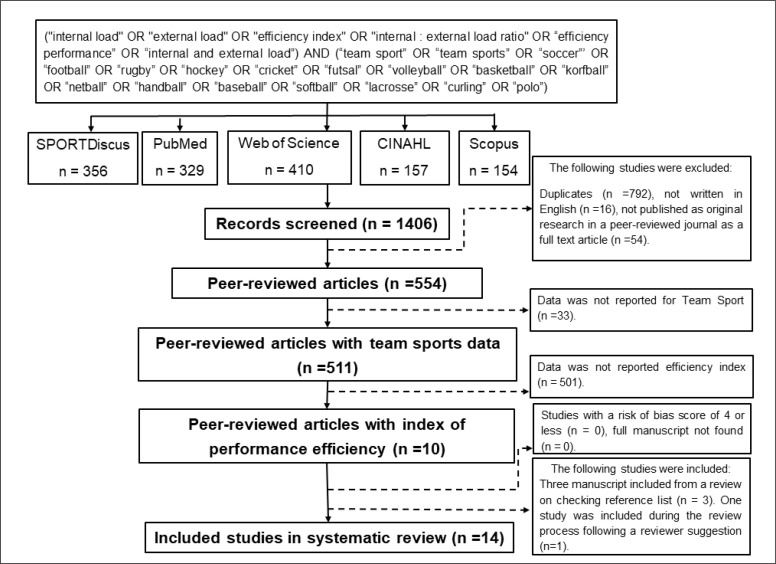
PRISMA diagram flow.

This review adopted the guidelines for Preferred Reporting Items for Systematic Reviews and Meta-Analyses (PRISMA) [23]. Five electronic databases (PubMed, Web of Science, Scopus, SPORTDiscus and CINAHL) were systematically searched up to March 2021. The authors created a Boolean search phrase to include search terms relevant to team sports athletes (population), internal load, external load, and the Eff_index_ (ratio between external and internal loads). Relevant keywords for each search term were delineated through a prior pilot search (screening abstracts, titles, keywords and full texts of previously known articles). The command line was composed of the following terms: ((“internal load” OR “external load” OR “efficiency index” OR “internal:external load ratio” OR “efficiency performance” OR “internal and external load”) AND (“team sport” OR “team sports” OR “soccer”’ OR “football” OR “rugby” OR “hockey” OR “cricket” OR “futsal” OR “volleyball” OR “basketball” OR “korfball” OR “netball” OR “handball” OR “baseball” OR “softball” OR “lacrosse” OR “curling” OR “polo”)) (see Figure 1).

### Study selection

The selection process and data extraction methods were completed independently by three researchers (AAL, CRC, and JGC). Abstracts were screened and reviewed by the same authors for identifying the potential eligible studies considering the inclusion criteria. The first author (AAL) retrieved and independently assessed the full text of the potential eligible studies. If any doubt arose, the disagreement was resolved through re-analysis until the consensus of all authors was achieved. The same procedures were applied to manual search within reference lists. In addition, one study during the review process was included following a reviewer suggestion (which also met the inclusion criteria).

### Eligibility criteria and selection process

Original research published in peer-reviewed journals, with language limitation but without date limitations, was eligible. Inclusion and exclusion criteria followed the PICOS [24] strategy detailed in Table 1. The general inclusion criteria were as follows:

**TABLE 1:** Inclusion and exclusion criteria (scope, PICOS and timeframe for follow-up).

Rule	Inclusion criteria	Exclusion criteria
**Participants**	Team sports athletes of any competitive level and sex.	Mixed results of team sports and individual sports.
**Interventions**	Participation in training or matches without time limitation.	Training in the gym (e.g, power training and weight lift).
**Comparators**	Without comparisons.	
**Outcomes**	Report any type of Eff_index_ calculation defined as a ratio between external and internal loads or the opposite ratio.	No reporting Eff_index._
**Study design**	Supervised randomized controlled trials, with either parallel or cross-over design. Non-randomized studies, Non-supervised intervention and/or comparators, Intervention or comparators supervised by professionals, Case studies, Cohort studies, Cross sectional.	Reviews, letters, opinion papers, meta-analysis and conference paper

1. The study was written in English.
2. The study was published as a full-text and original research paper in a peer-reviewed journal.
3. Data were reported only from team sports.
4. The participants were competitive athletes (defined as Olympic, international, professional, semi-professional or amateur, national, youth elite or division I collegiate).
5. The Eff_index_ should be described and used for monitoring.

When a paper was included, its reference list was subsequently checked by the first author (AAL) to search for other potential papers for inclusion [25].

### Data extraction

The first author (AAL) independently extracted the following information from the included full-text papers: (a) sample size and features (i.e., age, sex and competition level); (b) study characteristics and duration (i.e., weekly frequency and type/modality); (c) type of variables uses (i.e., TD, %HR_max_, TD/%HR_max_); (d) main results about the sensitivity of Eff_index_ to detect fatigue, recommendation of the most appropriate method to calculate Eff_index_ and level of correlation between Eff_index_ and physical fitness parameters. This information is presented in Table 4. A narrative synthesis of the results was subsequently performed.

### Quality assessment

The quality of all studies was evaluated using the risk of bias analysis described by Saw, et al. [26] (see Table 2), which has been used in previous systematic reviews [27, 28]. Scores were assigned based on how well each criterion was met, assuming a maximum possible score of 8 (low risk of bias). Studies with a risk of bias ≤ 4 were considered of poor quality and were subsequently excluded.

**TABLE 2:** Risk of bias assessment criteria.

	Criteria	Definition	Scoring		
			0	1	2
A	Peer reviewed	Study published in a peer-reviewed journal	No	Yes	-
B	Number of participants	Number of participants included in study findings	< 5	6–30	> 31
C	Population defined	Age, sex, sport, and time experience (or level) were described	No	Partly	Yes
D	Experimental design	Experimental design of the study period was described and replicable	No	Partly	Yes
E	Performance efficiency index	The efficiency index parameters were fully described	No	Yes	-

## RESULTS

The initial search returned 1,406 articles (see Figure 1). After the removal of duplicated articles (n = 792), a total of 614 studies were retained for full-text screening. Following eligibility assessment, studies with a risk of bias score ≤ 4 were considered of poor quality and were subsequently excluded (see Table 2). During the revision of the reference lists, only 3 studies met all the inclusion criteria and were also included in the systematic review. Moreover, one study was included during the review process following a reviewer suggestion. Therefore, a final pool of 14 studies were included in this systematic review.

### Characteristics of the studies and risk of bias

The pooled sample included 349 participants, only male athletes having being found, with an age of 23 ± 3 years. The sample group of seven studies was composed of elite professional athletes (50.0%), in six it was composed of non-elite athletes (43%), and one study did not report the competitive level (7%). Soccer players represent 59% of the sample, while 20% were rugby players, 10% were Australian football players, 7% were hurling players, and 4% were basketball players. The studies selected in this review mainly included field-based team sports [9, 14, 22, 29], and one study was related to an indoor-based team sport [30]. All the included studies had a low risk of bias, with a score > 4 (see Table 3). The average bias score for the studies was 7 (range 6–8).

**Table 3:** Risk of bias assessment criteria of each study

ARTICLE	A	B	C	D	E	TOTAL
Akubat et al. [31]	1	1	2	2	1	7
Kempton et al. [14]	1	1	2	2	1	7
Suarez-Arrones et al. [7]	1	1	1	2	1	6
Bucheit et al. [32]	1	1	2	2	1	7
Gallo et al. [11]	1	2	2	2	1	8
Malone et al. [22]	1	1	1	2	1	6
Torreno et al. [9]	1	1	2	2	1	7
Akubat et al. [10]	1	1	2	2	1	7
Delaney et al. [33]	1	2	2	2	1	8
Fox et al. [30]	1	1	2	2	1	7
Malone et al. [29]	1	2	2	2	1	8
Grünbichler et al. [21]	1	1	2	2	1	7
Reinhardt et al. [12]	1	2	2	2	1	8
Taylor et al. [13]	1	1	2	2	1	7

### Main results

A wide variety of combinations of external and internal load parameters were found in five different team sports (Table 4). To facilitate the understanding of Eff_index_ calculations, the running speed above 13 km/h was classified as high speed running (HSR), the TRIMP was considered any method based on the product of the time spent in different HR zones, and the quantification of speed changes with different thresholds was defined as accelerations. The details of each Eff_index_ calculation are described in Table 4.

**TABLE 4:** Detailed description of included studies

Article (1^st^ Author) and experimental design	Population (n,level, sex, age)	Study duration	Type of external load	Type of internal load	eff_index_ calculation	Main findings
Akubat et al. [31] Correlational study	10 soccer players, amateur, male 20 ± 1 years.	2 weeks	TD and HSR (> 15 km/h)	_i_TRIMP based on individual’s exponential heart rate-blood lactate profile	_i_TRIMP:TD; _i_TRIMP:HSR. During 30 min of Ball-Sport Endurance andSprint Test.	The _i_TRIMP:HSR was significantly correlated with _v_OBLA (r = 0.65; p = 0.04) and TD: _i_TRIMP with _v_LT (r = 0.69; p = 0.03). However, VO_2_max showed trivial to small correlation with these ratios. The results of this study suggest the use of these ratios in the assessment of aerobic fitness.
Kempton et al. [14] Prospective single cohort	18 rugby players, elite, male, 24.2 ± 3.6 years.	1 season	TD and Hsr (> 14,4 km/h)	Banister´s trimp	TD: TRIMP and HSR: TRIMP. During full games.	The Eff_index_ for both TRIMP: HSR and TRIMP:TD ratios was greater in the first 10-min of each half compared to later match stages (p < 0.001).
Suarez-Arrones et al. [7] Prospective single cohort	30 Soccer players, elite, male, not reported.	2 seasons	Mean speed in m·min^-1^	Mean exercise intensity in %HR_max_	Mean speed: % HR_max_. During the first half games.	The measures among position-specific players indicated that those with less overall running performance during matchplay showed the worst Eff_index_.
Buchheit et al. [32] Prospective single cohort	12 Soccer players, elite, male, 24.6 ± 5.3 years.	8 days	Mean speed in m·min^-1^	sRPE	sRPE: mean speed in m·min^-1^. During training sessions.	The sRPE: m·min^-1^ ratio was reduced throughout the training days (1st to 8th day).
Gallo et al. [11] Prospective single cohort	36 Australian football players, elite, male, 22 ± 2.5 years.	10 weeks	Mean speed in m·min^-1^, HSR (individual speed threshold range of 16.9 -19.7 km/h), PL per min and PL_slow_ per min	sRPE	Mean speed in m·min^-1^: sRPE; HSR:sRPE; PL:sRPE; PL_slow_:RPE. During training sessions.	The Eff_index_ mean speed:sRPE (p < 0.025) and PL_slow_:RPE (p < 0.001) were significantly impacted by pre-training wellness questionary Z-scores.
Malone et al. [22] Correlational study	25 Hurling players, not reported, male, 24 ± 4 years.	3 weeks	TD, HSR (≥ 17 km/h) and HSR (≥ 22 km/h)	_i_TRIMP based on individual’s exponential heart rate-blood lactate profile	HSR (≥ 22 km/h): iTRIMP HSR (≥ 17 km/h): iTRIMP TD: iTRIMP. During specific simulated match play.	The Eff_index_ was correlated with fitness measures, i.e. association between TD: iTRIMP and vOBLA, r = 0.56; TD: iTRIMP and VO_2_max, r = 0.52. External load only showed limited correlation.
Torreno et al. [9] Prospective single cohort	26 Soccer players, elite, male, 27.3 ± 3.4 years.	2 seasons	Mean speed in m·min^-1^	Mean exercise intensity in %HR_max_	Mean speed: % HR_max_. During full games.	The soccer players that showed higher overall match activity profiles had the highest Eff_index_. The Eff_index_ during the match was 1.3 ± 0.2 with substantial differences between first and second halves (1.4 ± 0.2 vs. 1.3 ± 0.2, respectively).
Akubat et al. [10] Correlational study	10 Soccer players, amateur, male, 20 ± 1 years.	2 weeks	TD, HSR (> 15 km/h), PL, MMP and HP (> 20 W.kg^-^)	_i_TRIMP based on individual’s exponential heart rate-blood lactate profile	TD:_i_TRIMP; HSR: _i_TRIMP; PL: _i_TRIMP; MMP: _i_TRIMP and HP: _i_TRIMP. During 30 min of Ball-Sport Endurance and Sprint Test. (BEAST90mod)	The Eff_index_ in rested conditions, showed large relationships with measures of fitness. The largest relationship was 0.69 (TD: iTRIMP), for vLT and 0.67 (HP: iTRIMP) for vOBLA. When the players are under fatigue conditions or not fully recovered there were moderate changes in some ratios as TD: iTRIMP, PL: iTRIMP and MMP: iTRIMP; and the relationships with fitness became weaker.
Delaney et al. [33] Prospective single cohort	38 Rugby players, elite, male, 23 ± 3 years.	50 days	Mechanical work, Impulse, Metabolic work, Hp distance, Acc/Dec load, HSR and TD.	sRPE and TRIMP based on banister model	sRPE or TRIMP: Mechanical work; Impulse; Metabolic work; Hp distance; Acc/dec load; HSR; TD. During training.	The Eff_index_ was considered appropriate for tracking individual responses to a pre-season. The appropriate variables considered for Eff_index_ calculation were TRIMP integrated with Acc, HSR, metabolic work, and mechanical work.
Fox et al. [30] Prospective single cohort	15 Basketball players, semiprofissional, 20.4 ± 4.5 years.	9 weeks	PL	sRPE and SHRZ based on Edwards model	sRPE: PL and SHRZ:PL. During training and competition.	A higher sRPE:PL ratio was observed in competition compared with training situations. However, SHRZ:PL ratio showed no significant result.
Malone et al. [29] Prospective two teams cohort	48 Soccer players, elite, male, 25.3 ± years.	1 season	TD, HSR (> 19.8–25.2 km/h), PL and PL_slow_	sRPE	TD:sRPE, HSR:sRPE, PL: sRPE, PL_slow_:sRPE. During training sessions	A reduction in wellbeing resulted in a negative impact in Eff_index_. The wellbeing Z-score of −1 resulted in −0.49 ± 0.12 m.min^−1^, −1.20 ± 0.08 m.min^−1^, −0.02 ± 0.01 AU min^−1^ in TD: sRPE, HSR: sRPE and PL_slow_: sRPE respectively.
Grünbichler et al. [21] Prospective single cohort	14 Soccer players, second league, male, 22.6 ± years.	13 weeks	ED	TRIMP_MOD_	ED: TRIMP_MOD_During full games.	Eff_index_ was negatively influenced by time duration of the session of the day before the match (β = -.216, p = .007). The training loads assessed during the days before a match were able to predict match Eff_index_.
Reinhardt et al. [12] Prospective cohort and correlation study	55 Soccer players, sub-elite, male, 24.6 ± 3.7 years.	3 seasons	Mean speed in m·min^-1^ and mean of speed multiplied by number of Acc (> 2 m/s^2^)	Mean exercise intensity in %HR_max_	Mean speed: %HR_max_; mean of speed multiplied by number of Acc: %HR_max_ (PI). During full games.	Eff_index_ among tactical positions displayed differences and the two types of Eff_index_ calculation were reduced in the 2^nd^ half. PI underwent less influence of distance covered by walking and jogging than mean speed:%HR_max_. Eff_index_ calculation based in PI equation was considered more adequate to detect fatigue.
Taylor et al. [13] Correlational study	12 rugby players, academy, male, 17.6 ± 0.44 years.	3 weeks	TD, PL, MMP, HRS (> 15 km/h), HSR (> 18 km/h), iHSR	_i_TRIMP based on individual’s heart rate-blood lactate profile	TD: iTRIMP; PL: iTRIMP; MMP: iTRIMP, iHSR: iTRIMP, HSR (> 15 km/h): iTRIMP, HSR (> 18 km/h): iTRIMP. During three exercise protocols.	Reliability of Eff_index_ results were described. All Eff_index_ calculation presented large to very large associations with vLT and vOBLA in the three exercise protocols. However, VO_2_max demonstrated small to moderate association with the ratios in the three exercise protocols used. TD: _i_TRIMP showed a similar reliability when compared to other more complex external load measures such as PL and MMP.

Acc = Acceleration; Dec = Deceleration; ED = Equivalent distance; HP = Distance covered at high metabolic power; HSR = High speed running; iHSR = Individual high speed running; _I_TRIMP = Individual training impulse; MMP = Mean metabolic power; vOBLA = Velocity at onset of blood lactate accumulation; %HR_max_ = Percentage of maximum heart rate; PI = Performance index; PL = Player load; PL_slow_ = Player load slow; _S_RPE = session Rating of perceived exertion; SHRZ = Summated heart rate zones based in Edwards model; TD = Total distance; VO_2_max = maximal oxygen uptake; TRIMP_MOD_ = Modified training impulse; vLT = Velocity at lactate threshold.

Ten studies showed Eff_index_ as an external-to-external load ratio and four studies as the opposite [30–33]. Regarding the internal load, five studies calculated Eff_index_ using sRPE [11, 29, 32, 33], 11 studies calculated Eff_index_ with HR-derived variables (Table 4), with two of them using also both variables [30, 33]. The HR-derived variables were percentage of maximum HR [7, 9, 12], individual training impulse (iTRIMP) [10, 13, 22, 31], TRIMP model of Banister [14, 33], TRIMP_MOD_ model of Stagno [21] and summated heart rate zones based on the Edwards model [30]. Regarding the external load, the Eff_index_ has been calculated using TD, HSR [7, 10], individual high speed running (iHSR) [13], Player Load, mean metabolic power, distance covered at high metabolic power [10], ED [34], accelerations [12], and Player Load Slow [29].

The results described included: 1) the correlation of Eff_index_ with physical fitness assessed during different exercise protocols, with this correlation tending to be large with velocity at onset of blood lactate accumulation (vOBLA) and weaker with maximal oxygen uptake (VO_2_max) [12]; 2) the effect of fatigue during matches on Eff_index_ [9, 14]; 3) the impact of a period of workload on Eff_index_ [11, 21, 29, 32, 33]; 4) calculation of Eff_index_ using accelerations multiplied by TD and divided by %HR_max_, which was called the performance index (PI) [12]; 5) the characterization of different tactical positions [7, 12], exercise protocols [13, 30] and competition [30] through Eff_index_; and 6) the description of validity and reliability of different Eff_index_ calculations in three exercise protocols [13].

The Eff_index_ has been recognised by all included studies as an important tool to monitor individual physical fitness status following its association with physical tests and fatigue (Table 4). Eff_index_ was preferred for physical fitness monitoring over the external load alone [22]. The monitoring of Eff_index_ of soccer players during matches has been shown to be higher in tactical positions characterized by elevated external loads [7, 12]. Most studies have used TD or the mean speed as external loads, with the TD:iTRIMP showing high reliability during sprint interval training (standard error of measurement = 7%) [13]. Another exercise protocol based on a continuous shuttle run test demonstrated a lower level of within-subject reliability (standard error of measurement = 16%). The use of accelerations to calculate Eff_index_ resulted in different values when comparing the use of only TD for its calculation (r^2^ = 0.56) [12]. The result of Eff_index_ calculated with accelerations entailed less influence of tactical positions and distance covered at low speeds [12].

## DISCUSSION

This review aimed to provide a better understanding of Eff_index_ as a tool to evaluate physical fitness status in team sports. The main findings of this review were: 1) the association of Eff_index_ with physical fitness; 2) the change of Eff_index_ scores according to the exercise protocol performed [13]; 3) the identification of TD divided by some HR-derived variables as a common Eff_index_ calculation; 4) and the existence of initial evidence suggesting that using acceleration parameters could increase the validity of Eff_index_ [12].

The assessment of internal and external load parameters has been done separately in team sports [4, 14, 15]. This, in turn, can be influenced by contextual factors such as opponent level and tactical performances [18] and, therefore, compromise the interpretation of fatigue effects on performance. The evidence that Eff_index_ is associated with physical fitness status is based on studies that describe the association between Eff_index_ and aerobic parameters of performance (i.e. vOBLA and vLT) when assessing hurling players [22], rugby players [13] and soccer players during specific exercise protocols in rested [10, 31] and fatigued conditions [10]. The correlation of the ratios with the submaximal aerobic parameters tended to be stronger than with maximal parameters (i.e. VO_2_max). Two studies found trivial to moderate correlations with VO_2_max [13, 31] and only one found a large correlation with VO_2_max [22]. This latter study used a single exercise protocol whilst the other studies used four different exercise protocols with a larger sample comprising two different sports. These different exercise protocols and samples may suggest that Eff_index_ changes could be mostly explained by submaximal aerobic parameters.

The investigation of fatigue and fitness status were the main objectives of some studies included in this review. The first research included was a correlational study from 2014 [31]; however, two previous studies which investigated the impact of fatigue on Eff_index_ were excluded because their samples were of referees [35, 36]. Therefore, it is important to note that the first evidence of Eff_index_ was first published with soccer and rugby referees in 2012 and 2013, respectively. The study of Barbero-Alvarez, et al. [35] found a reduction of Eff_index_ at the end of the match, and the study of Suarez-Arrones, et al. [36] showed a lower Eff_index_ in the last game of three consecutive games. These results are in agreement with those from the studies performed with athletes included in the current review. Thus, Eff_index_ has been considered sensitive to track the fatigue development over a match [9, 12, 14], adequate to identify a negative wellbeing status [11, 29], and valid to track individual responses during a period of workload [32, 33]. However, Eff_index_ may demonstrate a lower association with fitness status when the athletes are in a fatigued condition [10]. Moreover, a different response in Eff_index_ could be observed depending on the group of athletes examined, as previously discussed [37]. Therefore, it is important to consider individual data instead of average group responses.

Eff_index_ has been evaluated during both training sessions and matches [21, 30, 33]. For instance, basketball matches exhibited more internal load per external load performed than during training sessions using sRPE:PL [30]. However, when this ratio is displayed in the opposite manner [30, 31], the interpretation of the results would be adjusted to allow appropriate comparisons. Thus, while the increase of the internal-to-external load ratio would indicate the reduction of fitness status, on the other hand, the increase of the external-to-internal load ratio would be related to an augmented fitness status. Considering different types of training sessions, one study reported different reliability values among exercise protocols when using Eff_index_ [13]. Thus, the reliability found in a continuous shuttle run test (standard error of measurement of 16%) was not as good as that demonstrated in sprint interval and small-sided game sessions (standard error of measurement of 7% and 10%, respectively) [13]. With respect to these results, the selection of specific training sessions should be considered to assess fitness status while using Eff_index_. Of note, small-sided games are considered a highly specific and habitual type of training in team sports, thus incorporating sport skills at sufficient intensities to improve aerobic adaptations [38]. Therefore, its use could be highly recommended to measure Eff_index_.

We found different ways to calculate Eff_index_, with the use of TD as the most common external load parameter, and parameters derived from HR (i.e., TRIMP and %HR_max_) as the most frequent internal load parameter. However, three recent studies have suggested the use of an acceleration parameter to perform the Eff_index_ calculation [12, 21, 33]. Two of these studies indicated accelerations divided by TRIMP [33] and the equivalent distance parameter (including accelerations) divided by TRIMP [21], as sensitive enough for tracking individual responses to a training load. Previously, Reinhardt et al.[12] compared two methods, TD:%HR_max_ and TD multiplied by the number of accelerations:%HR_max_, and found that the use of the second equation entailed less influence of the distance covered by walking and jogging. The use of an acceleration parameter to calculate Eff_index_ in team sports is therefore recommended, because such sports are characterized by frequent changes of speed, with a high number of accelerations per match [2]. Furthermore, the number of accelerations can discriminate the competitive level of athletes [39]. On the other hand, we observed only four studies that used the simpler TD:sRPE calculation. Despite the low number of studies using this ratio, it has shown high sensitivity to detect changes in fitness status. Thus, TD:sRPE, when compared with other complex ratios, could be a simpler but valid tool to monitor fitness in team sports.

Physical fitness status is a complex concept, which can be better understood when a positive influence on performance is observed [25]. On the other hand, fatigue can occur rapidly and thus negatively influence physical performance [25]. Based on these concepts and the reviewed studies, there are several aspects to be investigated in future studies, including the evaluation of different competitive sports and levels, the influence of different environmental conditions and samples, including female athletes, and the need for more correlational studies to identify the most appropriate Eff_index_ calculation in each team sport setting.

This review is not without limitations. First, the studies selected mainly included field-based team sports, with other sports such as futsal and handball not considered. However, the quality of the 14 studies included could be considered appropriate, with 604 studies excluded because of inappropriate criteria or low quality. According to these studies, we suggest that Eff_index_ has sufficient evidence to be considered as an important tool to assess physical fitness status in team sports. In this context, to assess physical fitness status during competitions, it is important to minimize the effect of contextual factors such as tactical performance and level of opponents [18]. The Sport Science staff can easily use this tool in an applied environment, allowing the evaluation of athletes on a daily basis, including periods of congested match play [40]. However, it is very important to consider the recorded data on an individual basis, along with other important information about the physical fitness to adjust individual training loads (e.g. TD:iTRIMP) [37, 41]. This would contribute to better training load monitoring in team sports.

## CONCLUSIONS

Based on the current results, we suggest the use of Eff_index_ as an important tool for the evaluation of team sports athletes in various settings (i.e. competition, training, and testing). There are different ways to calculate Eff_index_. The most common Eff_index_ calculation was dividing TD by HR-derived parameters, such as TRIMP. However, recent evidence suggests the use of an acceleration parameter (i.e. TD × accelerations/%HR_max_). On the other hand, the use of TD divided by sRPE could be a valid but simpler option to assess fitness status in team sports. It is important to consider individual data and the type of exercise protocols to appropriately evaluate the physical fitness status of team sport athletes with this important monitoring tool. More studies with indoor team sports and female athletes are warranted.

## Potential conflict of interest

Author Adriano Lima Alves was employed by the company Associação Chapecoense de Futebol and University Federal of Minas Gerais; Author João Gustavo Claudino was employed by the company Load Control and School of Physical Education and Sport University of São Paulo. Authors Eduardo Mendonça Pimenta was employed by the company University Federal of Minas Gerais. Author: Crislaine Couto Rangel was employed by the company Centro Universitário Metodista Izabela Hendrix. Author Francisco Teixeira was employed by the Universidade Federal do Triângulo Mineiro. Daniel Boullosa was employed by the company Instituto Integrado de Saúde, Universidade Federal de Mato Grosso do Sul.

The remaining authors declare that the research was conducted in the absence of any commercial or financial relationships that could be construed as a potential conflict of interest.
